# Recurrent and Progressive CAVB After Alcohol Septal Ablation for HOCM: Bridging Therapy With a Semi‐Permanent Pacemaker

**DOI:** 10.1002/ccr3.73350

**Published:** 2026-08-20

**Authors:** Enyuan Zhang, Le He, Henan Zhang, Jing Xu, Fengmin Lu, Dongyan Wu, Yitong Yin, Wei Ma

**Affiliations:** ^1^ Tianjin Medical University Tianjin China; ^2^ Heart Rhythm Center, Department of Cardiology, Chest Hospital Tianjin University Tianjin China

**Keywords:** alcohol septal ablation, complete atrioventricular block, hypertrophic obstructive cardiomyopathy, semi‐permanent pacemaker

## Abstract

In patients undergoing alcohol septal ablation for hypertrophic obstructive cardiomyopathy, complete atrioventricular block can be delayed and recurrent. A semi‐permanent pacemaker serves as an effective bridging therapy, allowing for extended monitoring of conduction recovery and may spare selected patients from permanent device implantation.

## 1 Introduction

Alcohol septal ablation (ASA) is a well‐established treatment for symptomatic hypertrophic obstructive cardiomyopathy (HOCM) that is refractory to medical therapy. While ASA effectively reduces left ventricular outflow tract (LVOT) gradients, it carries a significant risk of iatrogenic atrioventricular conduction disturbances, including complete atrioventricular block (AVB). AVB is characterized by independent atrial and ventricular activity resulting from anatomical or functional disorders of the conduction system. The incidence of AVB following ASA ranges from 9% to 22%, often necessitating permanent pacemaker (PPM) implantation [1, 2]. Risk factors for AVB include pre‐existing bundle branch block (BBB), advanced age, female sex, larger ethanol injection volumes, and transient high‐grade AVB during the procedure [3, 4]. However, AVB can occur unexpectedly in patients without baseline conduction abnormalities, as illustrated by our case of a patient with atrial fibrillation (AF) and no pre‐existing BBB who developed refractory AVB after ASA. This case highlights the unpredictable nature of conduction system injury following septal infarction and underscores the need for vigilant monitoring and individualized pacing strategies.

Complete AVB may result from physiologic, pathophysiologic, or iatrogenic causes, some of which are reversible [5]. ASA was introduced in 1994 as an effective treatment option and an alternative to surgical myectomy for patients with HOCM. ASA helps reduce LVOT obstruction and associated symptoms. ASA‐induced myocardial necrosis occurs in the basal septum, which is adjacent to the cardiac conduction system and is likely to cause conduction block [6].

This is the first report of complete AVB lasting more than four days, managed with bridging therapy using a semi‐permanent pacemaker (sPPM) following ASA.

## 2 Case History and Examination

A 64‐year‐old male patient with persistent atrial fibrillation was admitted with a 10‐day history of wheezing. In the context of severe left ventricular outflow tract (LVOT) obstruction (gradient 50–60 mmHg) and marked systolic anterior motion (SAM) of the mitral valve, this respiratory symptom was highly suggestive of pulmonary venous congestion, commonly referred to as “cardiac asthma.” This presentation is a frequently under‐recognized manifestation of heart failure in hypertrophic obstructive cardiomyopathy (HOCM), often mimicking primary respiratory diseases such as asthma exacerbation and thereby delaying targeted cardiac intervention. Acute and critical care clinicians should be aware of this “cardiac asthma” phenotype to avoid inappropriate bronchodilator therapy and prioritize LVOT gradient reduction.

Transthoracic echocardiography revealed HOCM, with a left atrial diameter of 48 mm, left ventricular diameter of 49 mm, left ventricular ejection fraction of 56%, and interventricular septal thickness ranging from 15 to 19 mm. The LVOT velocity measured 3.4–3.7 m/s, corresponding to an estimated transvalvular pressure gradient of 47–55 mmHg. Mild‐to‐moderate mitral regurgitation was present, along with prominent SAM of the mitral valve. A Grade III/VI holosystolic murmur was best heard at the left sternal border between the third and fourth intercostal spaces, radiating to the axilla and back.

**FIGURE 1:**
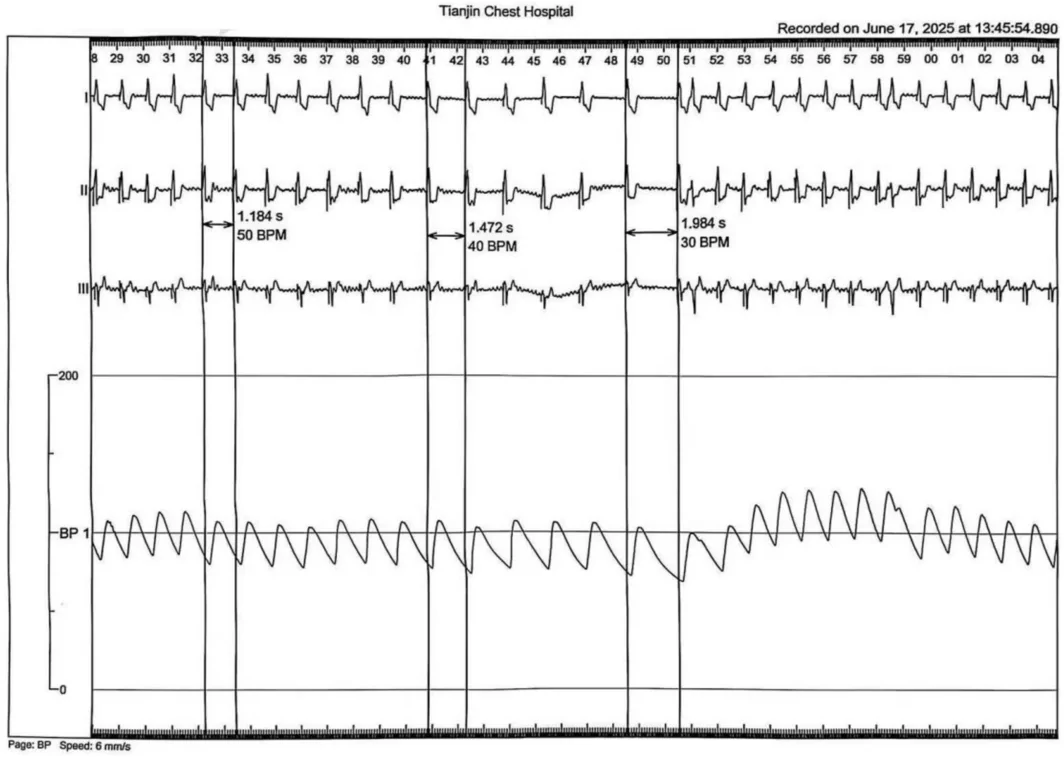
The ventricular escape rate dropped below 30 beats per minute after complete atrioventricular block during atrial fibrillation.

**FIGURE 2:**
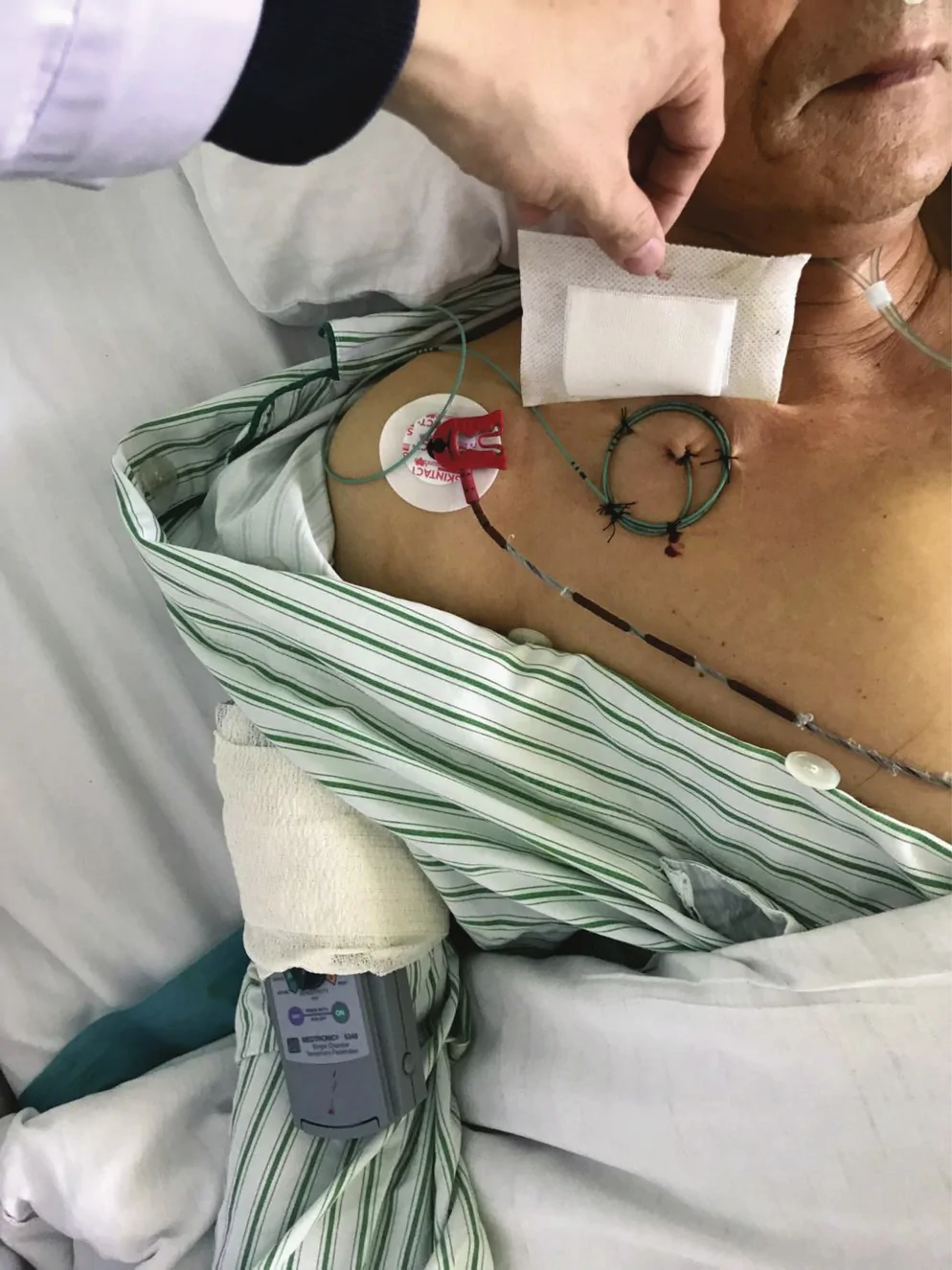
Semi‐permanent pacemaker was implanted via the right subclavian vein.

After comprehensive discussion, the patient elected to undergo alcohol septal ablation (ASA). No baseline bundle branch block was observed; however, RR intervals were notably prolonged. The LVOT pressure gradient was approximately 50–60 mmHg, and intracardiac echocardiography (ICE) confirmed SAM positivity. Under protection of a temporary pacemaker inserted via the femoral vein, 1.8 mL of absolute ethanol was injected into the first septal branch. During alcohol delivery, the patient developed complete atrioventricular block (CAVB) in the setting of atrial fibrillation (Videos 1, 2, 3). Ventricular escape rhythm fell below 30 beats per minute (Figure 1), while the LVOT gradient decreased to 10–15 mmHg and SAM resolved. No spontaneous ventricular conduction resumed after approximately one hour of observation; therefore, a semi‐permanent pacemaker (sPPM) was implanted via the right subclavian vein prior to transfer to the ward (Figure 2, Video 4).

**VIDEO 1:** Intraprocedural and post‐procedural electrophysiological recordings. Development of complete atrioventricular block during alcohol infusion into the first septal branch, with subsequent ventricular pacing dependency in the setting of atrial fibrillation. Video content can be viewed at https://onlinelibrary.wiley.com/doi/10.1002/ccr3.73350.

**VIDEO 2:** Intraprocedural and post‐procedural electrophysiological recordings. Development of complete atrioventricular block during alcohol infusion into the first septal branch, with subsequent ventricular pacing dependency in the setting of atrial fibrillation. Video content can be viewed at https://onlinelibrary.wiley.com/doi/10.1002/ccr3.73350.

**VIDEO 3:** Intraprocedural and post‐procedural electrophysiological recordings. Development of complete atrioventricular block during alcohol infusion into the first septal branch, with subsequent ventricular pacing dependency in the setting of atrial fibrillation. Video content can be viewed at https://onlinelibrary.wiley.com/doi/10.1002/ccr3.73350.

**VIDEO 4:** Intraprocedural and post‐procedural electrophysiological recordings. Fluoroscopic and electrocardiographic demonstration of semi‐permanent pacemaker implantation via the right subclavian vein. Video content can be viewed at https://onlinelibrary.wiley.com/doi/10.1002/ccr3.73350.

## 3 Methods (Differential Diagnosis, Investigations and Treatment)

This case report was prepared in accordance with the CARE guidelines. The patient's clinical data, imaging, electrophysiological recordings, and procedural details were retrospectively collected and analyzed. Investigations included transthoracic and intracardiac echocardiography, electrocardiography, continuous telemetry monitoring, Holter recording, and cardiac magnetic resonance imaging. Differential diagnoses considered for the presenting dyspnea included primary bronchial asthma, chronic obstructive pulmonary disease, and pulmonary embolism; these were excluded based on imaging findings and the characteristic “cardiac asthma” phenotype secondary to left ventricular outflow tract obstruction. Treatment decisions, including alcohol septal ablation and semi‐permanent pacemaker implantation, were made by the multidisciplinary heart team based on current guideline recommendations and individual patient factors.

## 4 Conclusion and Results

**FIGURE 3:**
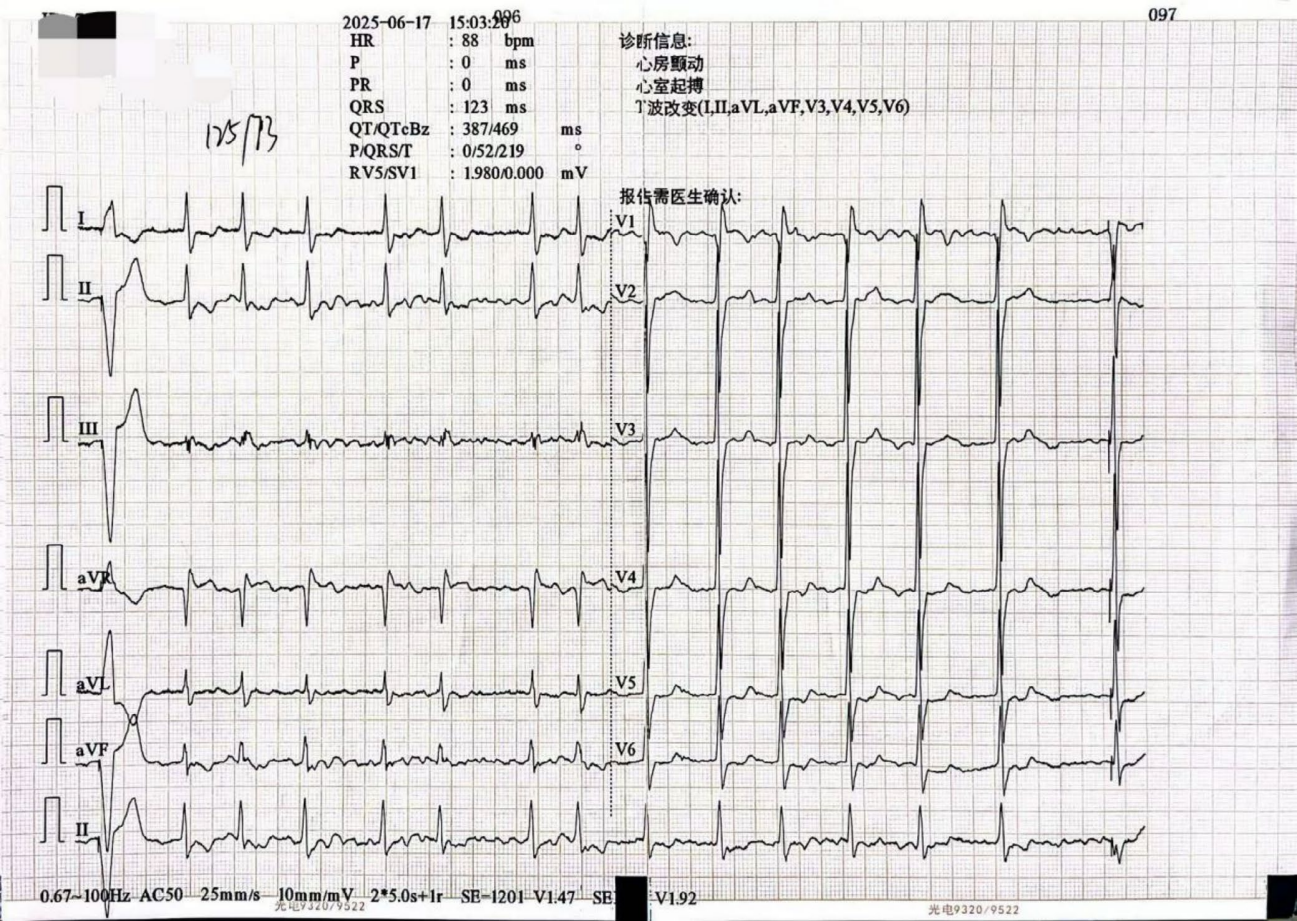
ECG showed a complete right bundle branch block after 4 h of complete atrioventricular block.

**FIGURE 4:**
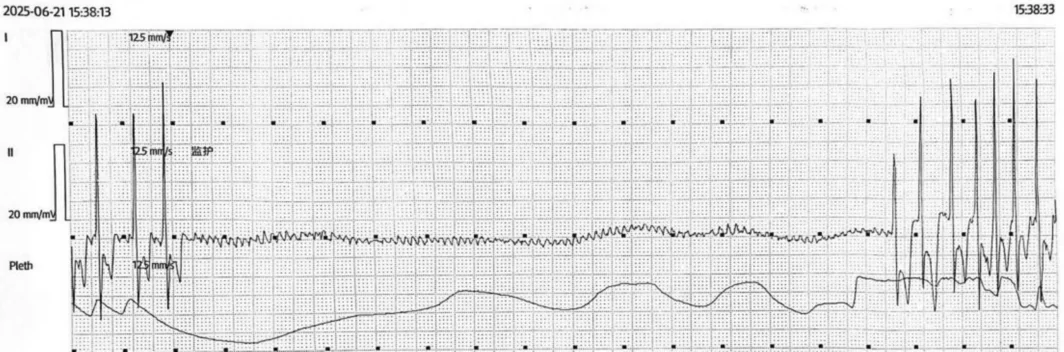
Episodes of extreme RR interval prolongation with ventricular pauses lasting up to 15 s.

Four hours after ASA, complete AVB resolved, and electrocardiography demonstrated a complete right bundle branch block pattern (Figure 3). Occasional ventricular pacing spikes were noted. On postoperative day (POD) 1, the patient developed a low‐grade fever despite antibiotic prophylaxis. The sPPM was electively removed on POD 3. However, on POD 4–24 h after pacing discontinuation—the patient experienced recurrent, unprovoked extreme RR interval prolongation and asystole, with ventricular pauses lasting up to 15 s (Figure 4). This abrupt recurrence highlighted the dynamic and unpredictable nature of post‐ASA conduction injury. Chest compressions and pharmacologic therapy were ineffective. During re‐implantation of the sPPM, the patient suffered repeated Adams–Stokes attacks. An urgent temporary pacemaker was placed via the femoral vein, followed by re‐implantation of a sPPM via the right subclavian vein after hemodynamic stabilization.

Transthoracic echocardiography on POD 6 showed persistent septal thickening (15–19 mm), but the LVOT velocity had decreased to 1.8 m/s, with a pressure gradient of 13 mmHg. SAM was no longer present, and LVOT obstruction was significantly relieved. By POD 9, the pacing rate was successfully reduced to 40 bpm. Holter monitoring revealed atrial fibrillation with a minimum heart rate of 49 bpm and an average of 84 bpm, confirming early spontaneous conduction recovery. The patient was discharged with the sPPM in situ.

One month later, the patient was readmitted for atrial fibrillation ablation (Video 5). Transthoracic echocardiography demonstrated residual septal thickness of 15–17 mm, LVOT velocity decreased to 1.2 m/s (gradient < 10 mmHg), and no prominent SAM. Post‐ablation Holter monitoring showed sustained sinus rhythm, with a minimum heart rate of 69 bpm and an average of 90 bpm, providing critical evidence that conduction function had fully recovered and allowing for safe removal of the sPPM. According to the 2021 ESC Guidelines on cardiac pacing, permanent pacemaker implantation can be avoided when the conduction disturbance is clearly reversible and recovery is documented. According to the ESC guidelines on cardiac pacing, permanent pacemaker implantation can be avoided when the conduction disturbance is clearly reversible and recovery is documented [7].

**VIDEO 5:** Intraprocedural and post‐procedural electrophysiological recordings. Intracardiac recordings during subsequent atrial fibrillation ablation procedure one month after alcohol septal ablation, with evidence of restored native conduction. Video content can be viewed at https://onlinelibrary.wiley.com/doi/10.1002/ccr3.73350.

## 5 Discussion

This case illustrates that CAVB can occur abruptly during ASA even in the absence of baseline conduction abnormalities. Although pre‐existing left bundle branch block (LBBB) is a well‐establishedpredictor [3], its absence does not preclude this life‐threatening complication. Karimianpour et al. [4] identified intraprocedural transient high‐grade AVB and postoperative PR interval prolongation (≥ 68 ms) as independent predictors of persistent AVB, reflecting dynamic electrophysiological changes during septal infarction. The underlying mechanism may involve ethanol‐induced necrosis of septal perforator arteries supplying the atrioventricular nodal region and proximal His–Purkinje system, irrespective of baseline conduction status.

Atrioventricular conduction disturbances following ASA are more common in elderly patients, with only 5% of younger patients requiring pacemaker implantation [8, 9]. Risk factors include pre‐existing LBBB, a PR interval greater than 200 ms, a high exercise‐induced pressure gradient, and advanced age [10, 11]. Our patient was relatively young, had no history of bradycardia or BBB, and the PR interval was not assessable due to AF. Notably, the septal thickness was not markedly increased, and the pressure gradient decreased immediately after the procedure. It is plausible that ethanol injection affected both the left and right bundle branches. Complete AVB typically occurs within the first 24 h after ASA [12]. The incidence of late complete AVB (beyond 48 h) is 8.9%, while between 4 days and 3 years it is 3.6%. No cases have been reported beyond 3 years [11]. Late AVB may result from progressive myocardial scarring and fibrosis, which are irreversible [13]. In our case, AVB occurred acutely and resolved quickly, only to recur abruptly on postoperative day 4 with a low escape rhythm. This was likely due to extensive ablation injury involving both bundle branches and severe post‐procedural myocardial edema.

**FIGURE 5:**
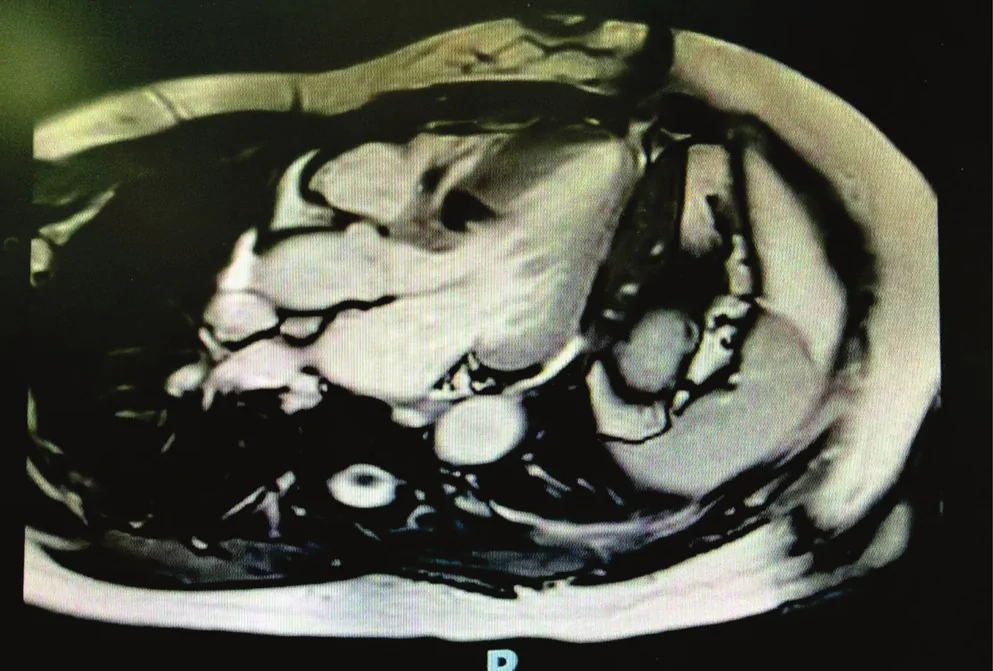
Cardiac MRI (gadolinium‐enhanced) showed focal late gadolinium enhancement (high signal intensity) in the interventricular septum 1 week post‐ablation, consistent with post‐ischemic myocardial scarring (arrows). This imaging finding correlates with ethanol‐induced necrosis of the septal perforator arteries supplying the conduction system.

The provided 1 week follow‐up cardiac MRI offers valuable insights. Focal abnormal signal intensity is noted in the interventricular septum at the site of alcohol ablation, consistent with post‐ablation myocardial injury in the acute phase. Focal late gadolinium enhancement in the interventricular septum corresponds to ethanol‐induced myocardial necrosis. This imaging correlate supports the hypothesis that conduction block resulted from direct injury to the septal perforator arteries supplying the atrioventricular node and proximal His‐Purkinje system—regardless of baseline conduction status (Figure 5). Prior to sPPM removal, Holter monitoring confirmed the absence of high‐grade AVB during daily activities, supporting the decision to explant the bridging device once conduction stability was evident.

This case highlights the controversy regarding permanent pacemaker (PPM) indication after ASA‐related AVB. While some guidelines suggest PPM implantation if procedure‐related AVB persists beyond 24–48 h, our experience argues for a more nuanced approach. In patients where AVB resolves initially but who have risk factors for late recurrence (e.g., extensive ablation, significant edema on imaging), a strategy of bridging therapy with an sPPM allows for extended monitoring of conduction recovery. This approach can spare selected patients from unnecessary permanent device implantation, as illustrated here, but necessitates vigilant monitoring protocols.

Consistent with the 2021 ESC Guidelines on cardiac pacing, permanent pacemaker implantation can be safely deferred—and ultimately avoided—when the conduction disturbance is clearly reversible and recovery is objectively documented during an adequate observation period [7]. Our case provides a practical example of how a semi‐permanent pacemaker facilitates such documentation, enabling a tailored, physiology‐guided pacing strategy.

AF is more common in female patients with HOCM and is associated with poorer outcomes following ASA [14]. In this case, AF likely exacerbated hemodynamic instability during AVB, necessitating prompt pacing support. AF independently increases the risk of atrioventricular nodal dysfunction due to structural remodeling, potentially sensitizing the conduction system to alcohol‐induced injury. Our case, with CAVB recurring on POD 4 following removal of temporary support on POD 3, strongly supports the recommendation for extended telemetry monitoring for at least 4–5 days post‐ASA, as suggested by prior studies.

Prophylactic temporary pacing is essential during ASA. Previous studies suggest that early permanent pacemaker implantation should be considered if procedure‐related AVB persists beyond 24 h [15]. However, after extended observation (e.g., one month), some patients may avoid permanent pacing. Kern et al. recommend extended telemetry monitoring for at least four days after ASA to detect late‐onset AVB [16]. In cases of recurrent AVB, transitioning to a sPPM improves stability and mobility while reducing risks associated with femoral access.

AVB following ASA can manifest late (more than 48 h post‐procedure) or recur after initial resolution, as demonstrated in this case. Schuller et al. [11] reported late AVB in 8.9% of patients, with occurrences documented up to three years after the procedure. Kern et al. [16] described AVB occurring 72 h post‐ASA without prior warning, highlighting that transient intraprocedural block does not ensure safety. The use of sPPM (e.g., leadless systems or extended temporary pacing) can provide support during recovery, reduce risks associated with femoral access (such as thrombosis and infection), and facilitate patient mobilization [2].

## Author Contributions

**Yitong Yin:** conceptualization, data curation, formal analysis, methodology, resources, software, writing – review and editing. **Le He:** conceptualization, data curation, methodology, software, validation, writing – review and editing. **Henan Zhang:** data curation, investigation, methodology, software, validation, writing – review and editing. **Enyuan Zhang:** conceptualization, data curation, formal analysis, investigation, methodology, project administration, resources, software, supervision, validation, visualization, writing – original draft. **Fengmin Lu:** data curation, investigation, project administration, supervision, validation, writing – review and editing. **Jing Xu:** conceptualization, formal analysis, methodology, software, visualization, writing – review and editing. **Wei Ma:** conceptualization, funding acquisition, project administration, resources, software, supervision, validation, writing – review and editing. **Dongyan Wu:** conceptualization, data curation, formal analysis, methodology, supervision, writing – review and editing.

## Funding

This study was supported by the Tianjin Health Science and Technology Project (TJWJ2022QN068), Integrated Traditional Chinese and Western Medicine, Tianjin Municipal Health Commission (12000023P098L0610072U), 2025 Clinical Research Funding Program of the Asian Society of Cardiac Electrophysiology (AHRA‐2025‐C‐[0015]) and the Tianjin Key Medical Discipline Construction Project (Specialty TJYXZDXK‐3‐017B).

## Ethics Statement

Ethical approval was granted by the Ethics Committee of Tianjin Chest hospital.

## Consent

Written informed consent was obtained from the patient for his participation in the study for the publication of the report.

## Conflicts of Interest

The authors declare no conflicts of interest.

## Data Availability

All raw data and code are available upon request.
